# Structural and functional analysis of cell adhesion and nuclear envelope nano-topography in cell death

**DOI:** 10.1038/srep15623

**Published:** 2015-10-22

**Authors:** Hyuk-Kwon Kwon, Jae-Hyeok Lee, Hyeon-Jun Shin, Jae-Ho Kim, Sangdun Choi

**Affiliations:** 1 Department of Molecular Science and Technology, Ajou University, Suwon 443-749, Korea; 2 Department of Materials Science and Engineering, Northwestern University, Evanston, Illinois 60208, USA

## Abstract

The cell death mechanisms of necrosis and apoptosis generate biochemical and morphological changes in different manners. However, the changes that occur in cell adhesion and nuclear envelope (NE) topography, during necrosis and apoptosis, are not yet fully understood. Here, we show the different alterations in cell adhesion function, as well as the topographical changes occurring to the NE, during the necrotic and apoptotic cell death process, using the xCELLigence system and atomic force microscopy (AFM). Studies using xCELLigence technology and AFM have shown that necrotic cell death induced the expansion of the cell adhesion area, but did not affect the speed of cell adhesion. Necrotic nuclei showed a round shape and presence of nuclear pore complexes (NPCs). Moreover, we found that the process of necrosis in combination with apoptosis (termed nepoptosis here) resulted in the reduction of the cell adhesion area and cell adhesion speed through the activation of caspases. Our findings showed, for the first time, a successful characterization of NE topography and cell adhesion during necrosis and apoptosis, which may be of importance for the understanding of cell death and might aid the design of future drug delivery methods for anti-cancer therapies.

Cell death is commonly categorized into two mechanisms; apoptosis and necrosis, depending on the diverse manner in which cell death is stimulated and the different biochemical and morphological characteristics that are presented for each process^1^,^2^. Alterations in cell death have been shown to be closely associated with developmental disorders, neurodegenerative diseases, and cancer^3^. Apoptosis is programmed cell death, mediated by the mitochondrial or intrinsic signaling pathway and the death receptor or extrinsic signaling pathway, resulting in cell shrinking and characteristic apoptotic morphological changes^4^. On the other hand, necrosis, also known as unprogrammed cell death, triggers morphological changes including cell or nucleus swelling and plasma membrane disruption^4^. Chan *et al.* previously reported that tumor necrosis factor induces receptor-interacting protein-mediated necrosis, in cysteine-aspartic protease-8 (caspase-8) knockout cells, in which they reported the presence of necrotic morphological characteristics, such as organelle swelling and plasma membrane disruption^5^. The progression of necrosis consists of the formation of a necrosome by receptor-interacting proteins, and the generation of reactive oxygen species, as well as other factors which have been shown to contribute to necrosis^6^. Recently, a new concept suggesting that necrosis also participates in programmed cell death, termed necroptosis, has been proposed, and studies on this mechanism and its characteristics have been performed^6^,^7^. However, the mechanism by which necrosis participates in necroptosis and the characteristic features of the process are not yet fully understood.

During apoptotic cell death, two characteristic stages, the first consisting of fragmentation of focal adhesion, the second involving nuclear envelope (NE) destruction, as well as nuclear DNA and protein fragmentation, occurring through the activation of the caspase-dependent pathway, are generally observed^8^.

With respect to the first stage, cell adhesion plays an important role in cell migration, growth, differentiation, and morphology. Cell adhesion is generally regulated by cell adhesion molecules, the extracellular matrix, cell junctions, and peripheral membrane proteins^9^. The cytoskeletal proteins assemble in order to generate mechanical force (cell stretching or fluid flow) for important cellular processes, such as establishment of cell adhesion and activation of signaling pathways^10^,^11^. A recent study in stem cell research showed that the adhesion molecule, β-catenin, is required for the formation of the mesendodermal germ layer and the differentiation of neuronal cells from embryonic stem cells (ESCs)^12^. Studies of cell death have shown that the activation of caspase-3 triggers the cleavage of the key factors of focal adhesion proteins, which are important for the control of cell behavior^13^, such as Crk-associated substrate (CAS) and focal adhesion kinase (FAK). Caspase-3 has also been reported to contribute to apoptotic morphological changes^14^,^15^. Moreover, multiple drugs such as zoledronic acid, vincristine, cytochalasin D, and paclitaxel may induce apoptosis by the caspase-induced destruction of cytoskeletal proteins such as α-tubulin and phalloidin^16^.

Secondly, in eukaryotic cells, the NE envelopes the nucleoplasm, which contains the genetic information of a cell, separating it from the cytoplasm, and has an important role in the control of nucleocytoplasmic transport of shuttle proteins and RNA factors^17^,^18^. The NE consists of a double-membrane, separately known as the inner nucleus membrane (INM) and outer nucleus membrane (ONM), and nuclear pore complexes (NPCs) are embedded in both membranes^19^,^20^. Intermediate filament proteins (type A, B, and C lamins) interconnect with the INM and DNA to provide nuclear shape, DNA stabilization, and NE structures^21^,^22^. The NPC is composed of multiple proteins, known as nucleoporins (NUPs), which control the bi-directional transport of diverse molecules from the nucleus and cytoplasm^17^,^23^,^24^. Previous studies have shown that DNA damage-inducing chemicals generated caspase activation and triggered cleavage of NPC proteins (e.g., NUP 93, 153, and 214) and INM proteins (e.g., lamina-associated polypeptide 2), during apoptosis^25^,^26^,^27^. The disruption of nuclear scaffold (also known as nuclear matrix) proteins, such as lamins and the NPC, is increased in apoptotic cell death^28^. A recent study showed that caspase mediated the induction of NE disruption during apoptosis, and the results were confirmed through fluorescence intensity, measured by confocal microscopy^29^. However, the changes that occur in cell adhesion and the NE, and how these alterations and characteristics correlate to necrotic and apoptotic cell death, are not clearly understood. Moreover, cell adhesion and NE topography are typically measured by protein expression levels and morphometric analysis; however, these methods are not ideal because of their indirect detection of effects and their limitation to partial observation.

Here, we demonstrate that necrosis and apoptosis regulate the characteristics of both cell adhesion and NE alteration, using a model of doxorubicin (DOX)- and etoposide (ETO)-induced DNA damage, in normal human kidney (HK-2) cells^20^,^30^. We also introduce a novel and direct application of the xCELLigence cell analysis system and carbon nanotube-attached AFM (CNT/AFM) probes, for cell adhesion and NE topography analyses.

## Results

### DOX completely induced necrosis, while ETO induced necrosis in combination with apoptosis

We investigated apoptotic and necrotic cell death, caused by DOX and ETO, by evaluating cell viability, caspase-3/7 activity, and Annexin V/propidium iodide (PI) staining. For cell viability analysis, DOX (1 μM) and ETO (50 μM) treatment were used to induce cytotoxicity in a time-dependent manner (half maximal inhibitory concentration; IC50 induced at 72 h) (Supplementary Fig. S1A). ETO-treated cells exhibited significantly higher caspase-3/7 activity and percentage of cells positively stained with PI (indicating necrosis) and Annexin V (indicating apoptosis) simultaneously, than control cells. On the other hand, DOX-treated cells exhibited a higher percentage of PI-positive cells than the control, and this increase was almost independent of caspase-3/7 activity and Annexin V-positive cells (Supplementary Fig. S1B, C). These findings suggest that DOX-treatment definitively induced necrosis, but ETO-treatment simultaneously induced necrosis and apoptosis (referred to as nepoptosis).

### Necrosis and nepoptosis generated cell swelling, but yield different effects on cell adhesion

**Figure 1:**
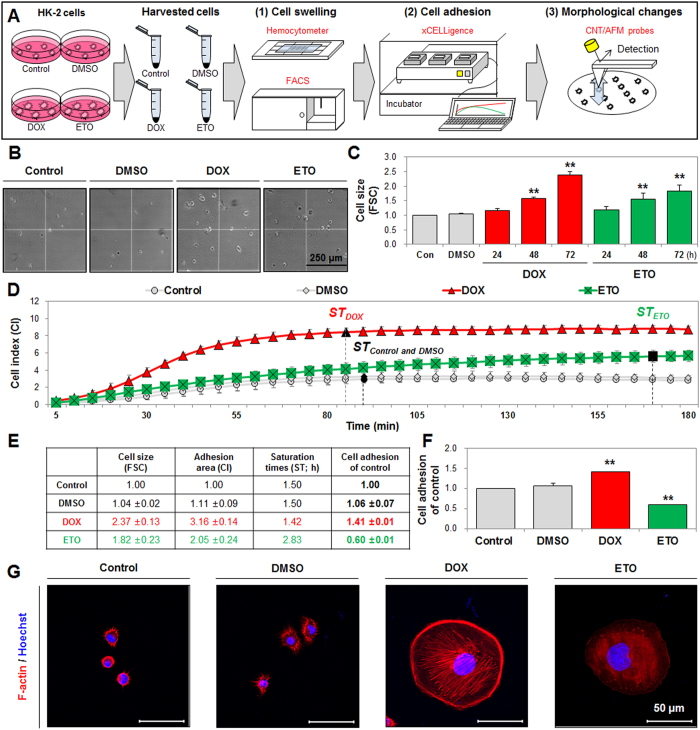
Doxorubicin (DOX) and etoposide (ETO) induced cell swelling, while differently regulating cell adhesion. (**A**) Schematic of necrosis and nepoptosis regulation of cell swelling, cell adhesion, and morphological changes measured using a hemocytometer, FACS, the xCELLigence system, and CNT/AFM probes. (**B**) DOX- and ETO-induced cell swelling were visually measured using hemocytometer analysis and detected by phase contrast microscopy (scale bar represents 250 μm) at 72 h. (**C**) DOX- and ETO-induced numerical cell size measured using FSC units of analysis for increasing times. (**D**) DOX- (red color) and ETO (green color)-treated cells were harvested after 72 h and cell adhesion was measured, including cell index (CI) and saturation times (ST) detected using xCELLigence for 10 s intervals and monitored for 3 h. Black closed diagram indicate saturation (±1.0%) times. (**E**,**F**) Table shows the cell size (FSC), adhesion area (CI), and saturation times (ST; h) at which cell adhesion was examined using computational analysis (described in results) compared to the control. The histogram shows cell adhesion levels calculated using the derived formula (described in results) for DOX- and ETO-treated cells for 72 h. (**G**) DOX-and ETO-treated cells were seeded in culture dishes for 3 h. Expression level of F-actin (red) was measured using immunofluorescence staining and detected by confocal microscopy. Hoechst 33258 (blue) was used for nuclear staining and scale bar represents 50 μm. All histograms represent statistical analysis (P-value of *P < 0.05, **P < 0.01).

In our experiments, we observed that DOX- and ETO-treatment of cells commonly resulted in an expanded cell adhesion area on culture dishes. Moreover, almost all DOX-treated cells attached to the dish, but a large number of ETO-treated cells remained in the culture supernatant, indicating a loss of cell adhesion capability (data not shown). Thus, we determined that necrosis and nepoptosis induced different alterations in the cells’ ability to adhere in culture. The measurement of alterations in cell size, adhesion, and morphology caused by necrosis and nepoptosis were confirmed by the use of a hemocytometer, fluorescence activated cell sorting (FACS), xCELLigence technology and CNT/AFM probe methods (Fig. 1A).

We demonstrated that cell size of DOX- and ETO-treated cells was visually increased, via analysis on a hemocytometer, when compared to the control cells (Fig. 1B). Using forward scatter light (FSC, indicative of cell size) analysis in FACS, we determined the induced FSC levels from 1 × 10^4^ cells (Fig. 1C). Subsequently, we investigated alterations in cell adhesion ability caused by DOX and ETO, using the xCELLigence system. The xCELLigence system is used for cell-based measurements for analysis, including cell viability, invasion, and migration, by electrical impedance (cell index [CI]) in real-time using a gold microelectrode pattern device^31^,^32^, but alterations in cell adhesion are not usually determined during cell death. Here, we suggested a new application of this method to measure changes in cell adhesion, and established a formula to calculate cell adhesion alteration caused by necrosis and nepoptosis. We harvested DOX- and ETO-treated cells at 72 h (only attached cells on culture dish; supernatant cells were removed), seeded them (1 × 10^5^/well) into devices, and used the xCELLigence system to measure CI and saturation time (ST) at 10 s intervals, for 3 h. Results from the xCELLigence system showed that the CI level was significantly higher (3.2-fold increase) in DOX-treated cells than in control cells, but the ST was not affected. The CI for ETO-treated cells was also higher (2.1-fold increase) than the control cells, and the ST was delayed (90 min to 150 min) (Fig. 1D). The CI value is indicative of the cell adhesion area, and the CI ratio represents the increase from the cell size (FSC) ratio. Accordingly, when the CI ratio value is divided by the FSC ratio value, a value greater than 1.0 indicates the extension of the cell adhesion area, and generation of cell swelling, but values less than 1.0 indicate a loss of cell adhesion. Acceleration or deceleration of ST indicates the increase or decrease in cell adhesion speed, respectively. Based on these assumptions, we established the following formula to calculate cell adhesion (with each value described as shown in Fig. 1E):


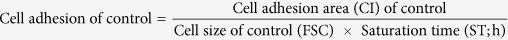


Once calculated, our results showed that DOX-treatment induced cell adhesion (1.4-fold change from control), with increased CI, while the ST point was not affected, indicating cell swelling. ETO-treated cells exhibited reduced cell adhesion (0.6-fold change from control), with decreased CI and a delayed ST point, indicating a loss of cell adhesion capacity (Fig. 1F). Similarly, DOX- and ETO-treated cell size was initially increased from the size of the control cells, at 0 min, and progressive cell swelling was observed, using real-time live-cell imaging analysis (Supplementary Fig. S2A and Movie S1). DOX-treated cells were almost fully attached to the culture dish, but attachment of ETO-treated cells was less than DOX-treated cells (Supplementary Fig. S2B and Movie S1). Analysis of diameter to adhesion cells using phase contrast microscopy showed that control and DMSO-treated cells appeared approximately 20 ~ 30 nm in diameter, but DOX- and ETO-treated cells appeared approximately 60 ~ 100 nm in diameter at 3 h (Supplementary Fig. S2B). When measured using confocal microscopy, control and DMSO-treated cells indicated lower cell spreading area (averagely control: 243.7 μm^2^ and DMSO: 230.9 μm^2^), and DOX- and ETO-treated cells indicated higher cell spreading area (averagely DOX: 8188.7 μm^2^ and ETO: 7269.6 μm^2^). The expression of cytoskeleton protein such as F-actin in the cytoplasm was observed in control, DMSO and DOX groups. However, ETO-treated cells showed significantly decreased expression levels when compared with DOX group (Fig. 1G). Thus, necrotic cell death induced the expansion of the cell adhesion area, but did not affect the speed of cell adhesion, while apoptotic cell death reduced the cell adhesion area and cell adhesion speed.

### Preservation of necrotic morphological changes during necrosis and nepoptosis

**Figure 2:**
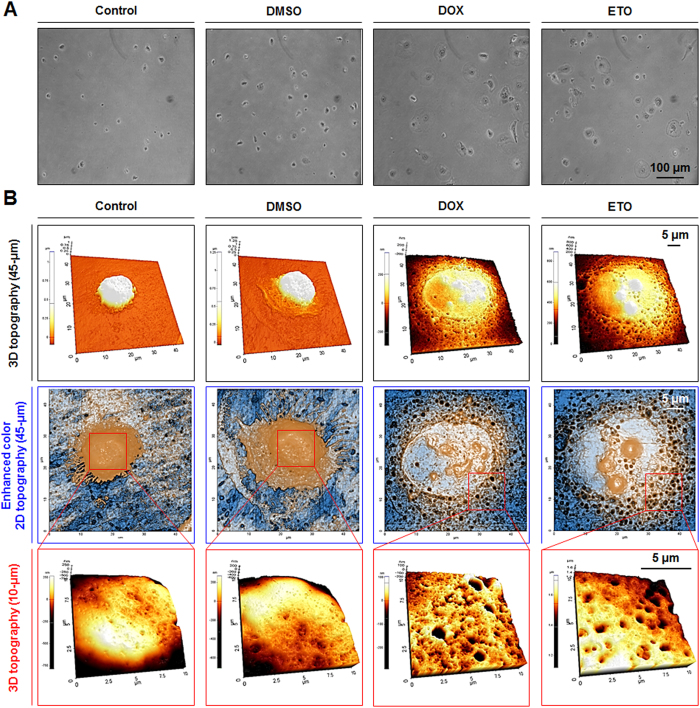
Doxorubicin (DOX) and etoposide (ETO) induced cell swelling and necrotic plasma membrane ruptures. (**A**) DOX- and ETO-treated cells were seeded in culture dishes for 3 h, and cell swelling was measured by phase contrast microscopy (scale bar represents 100 μm). (**B**) DOX- and ETO-treated cells were harvested and seeded on culture dishes for 3 h, after which topography changes in the plasma membrane were measured using CNT/AFM probes. Images represent the 3D topography (45- and 10-μm scale) and enhanced color topography (45-μm scale) using the XEI software, which is described in Supplementary Fig. S3.

Cell swelling is an important indicator of necrosis; another characteristic of necrosis induction is the rupturing of the plasma membrane. In our study, DOX- and ETO-treated cells also illustrated cell swelling and expansion of cell adhesion area compared with control and DMSO groups (Fig. 2A). Therefore, we examined plasma membrane topography of adherent DOX- and ETO-treated cells, using CNT/AFM probes. AFM has been identified as a reliable, convenient technique for high-resolution investigation of nanostructures and biological materials at a nanometer scale, and AFM image resolution is dependent on the tip sharpness. In a previous report, we demonstrated that the CNT/AFM probes fabricated by the Langmuir-Blodgett technique had high-resolution imaging capability, for visualizing nanostructures and biological materials, such as nanoporous alumina membranes and plasmid DNA, respectively, to accurately identify their morphological traits^33^. In our study, AFM images showed that HK-2 and DMSO-treated cells appeared to have a low number of ruptures in their plasma membrane, when collected with trypsin-EDTA, showing a only small amount of damage was caused by the collection technique. On the other hand, DOX- and ETO-treated cells were shown to definitively induce necrotic morphological changes as evidenced by cell swelling and the significant plasma membrane rupturing observed at the nanometer scale (Fig. 2 and Supplementary Fig. S3). In the roughness analysis on the plasma membrane at 2 μm scale, control and DMSO-treated cells appeared 37.5 nm and 31.2 nm roughness, respectively. However, DOX- and ETO-treated cells significantly increased roughness to 53.2 nm and 57.0 nm, respectively. Furthermore, DOX- and ETO-treated cells decreased cell height (DOX: 425.6 nm and ETO: 408.0 nm) compared with control (1021.0 nm) and DMSO-treated (1058.0 nm) cells (Supplementary Fig. S3). Thus, in necrosis, a reduction of cell height and induction of cell adhesion area as well as increased plasma membrane roughness were observed.

### Necrosis and nepoptosis generated nucleus swelling and altered nucleus morphology

In our experiments, DOX- and ETO-treated cells exhibited an increase in nucleus size, which is a typical morphological characteristic observed during necrotic cell death. Therefore, we used nucleus targeting dyes, Hoechst 33258 and PI, to assess nucleus area, and quantitatively measured the sizes of the nuclei using a Cellomics ArrayScan HCS reader system and analyzing at least 200 cells per well. This computerized system automatically provides various analyses, including cell viability, protein expression, and morphological changes, based on the acquired nucleus area and fluorescence intensity parameters. When compared to the control cells, the area of the nuclei of DOX- and ETO-treated cells, as well as the amount of blue staining from the Hoechst 33258 dye (visual representation of nucleus swelling by confocal microscopy analysis) was dramatically increased (Supplementary Fig. S4A,D). In addition, Hoechst 33258 and PI fluorescence intensities were higher in the nuclei of DOX- and ETO-treated cells than control cells, but relatively, the intensity was lower in the nuclei of ETO-treated cells when compared to that of the DOX-treated cells (Supplementary Fig. S4B,C).

**Figure 3:**
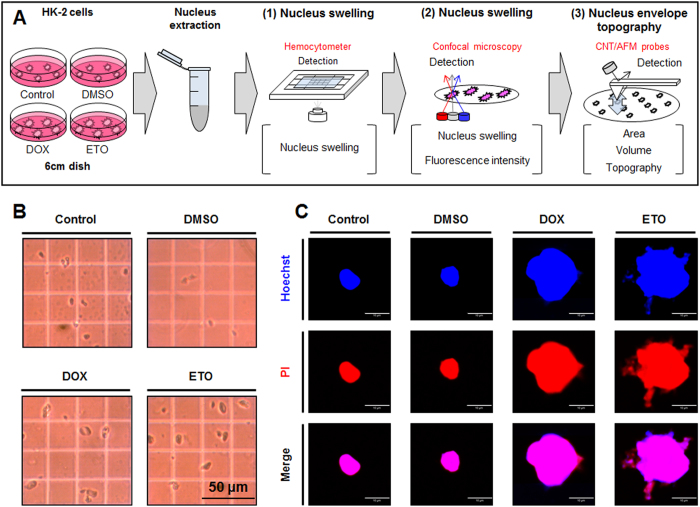
Doxorubicin (DOX) and etoposide (ETO) induced nucleus swelling, while differently affecting nucleus morphology. (**A**) Schematic of nucleus swelling and nuclear envelope topography measured using a hemocytometer, confocal microscopy, and CNT/AFM probes systems. (**B**) DOX- and ETO-treated cells were harvested and the nuclei were extracted. Nuclear extracts were seeded on a hemocytometer and visually measured using phase contrast microscopy (scale bar represents 50 μm). (**C**) Nuclear extracts were seeded on coverslips for 15 min and stained with nucleus targeting dies such as Hoechst 33258 and PI dyes that were detected using confocal microscopy. Representative images shown are the merged images for staining with Hoechst 33258 (blue color) and PI (red color; scale bar represents 10 μm).

Based on these observations, we hypothesized that necrosis and nepoptosis regulate nucleus morphology and NE topography in different manners, and we used three measurement methods; hemocytometer analysis, confocal microscopy and CNT/AFM probes, to directly evaluate this (Fig. 3A). Preferentially, we used NE-PER Nuclear and Cytoplasmic Extraction Reagents Kit for accurate and secure nucleus extraction. The extraction kit contained Cytoplasmic Extraction Reagent I and II, and Nuclear Extraction Reagent. Cytoplasmic Extraction Reagent I causes cell membrane disruption but not disruption of the NE, while Cytoplasmic Extraction Reagent II inhibits the activity of Cytoplasmic Extraction Reagent I. The nuclear extracts were seeded onto a hemocytometer to visualize nucleus swelling that was definitively shown to result in increased nucleus size, when compared to the control on an equal scale (Fig. 3B). Interestingly, we observed that when extracting the nuclei of ETO-treated cells a lot of debris and leaked DNA were present in the supernatant from the nuclear extraction, when compared to the supernatant from DOX-treated cell nuclear extraction, in which neither debris nor leaked DNA were observed. Moreover, we observed that extracted nuclei attached easily to the coverslip and cell culture dish. For that reason, the extracted nuclei were seeded on coverslips and cell culture dishes for a short time (15 minutes), in order to remove the debris, then washed with PBS for at least 3 times, for a complete removal of debris. Nuclear extracts were put into culture dishes and stained with nucleus targeting dyes (Hoechst 33258 and PI) for confirmation of the nucleus. The extracts exhibited nuclear swelling and co-localization (pink color) of the Hoechst 33258 (blue color) and PI (red color) dyes (Fig. 3C). The nuclei of DOX-treated cells were round in shape with a distinct nuclear boundary, while the nuclei of ETO-treated cells were an irregular shape, and DNA leakage from the nuclei was observed.

### Nepoptosis induced NE rupturing and DNA leakage from the nuclei

**Figure 4:**
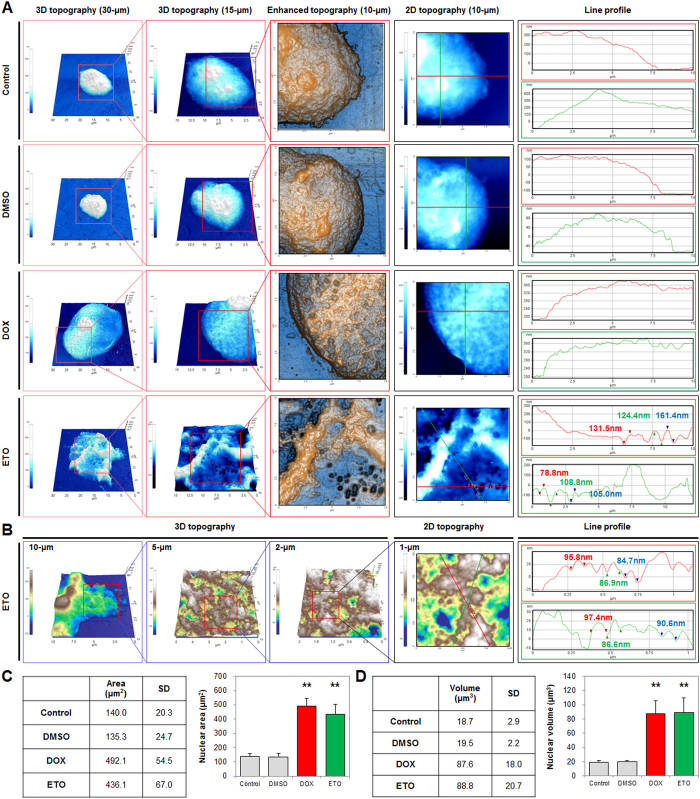
Doxorubicin (DOX) and etoposide (ETO) induced nucleus swelling and changes in area and volume, while ETO triggered nuclear envelope rupturing and DNA leakage. (**A**) Nuclear extracts were seeded on culture dishes for 15 min, and the nuclear envelope topography of fixed nuclei was measured using a CNT/AFM probe system. Images shown represent the 3D topography (30- and 15-μm scale), enhanced color topography (10-μm scale), and line profiles (10-μm scale). See also Supplementary Fig. S5. (**B**) Images show the DNA released in nuclei with 3D topography (2- to 10-μm scales), 2D topography (1-μm scale), and line profiles. (**C**,**D**) Table and histograms show the average nucleus area (μm^2^) and nucleus volume (μm^3^) analyzed using XEI software in 10 nuclei (statistical analysis P-value of *P < 0.05, **P < 0.01).

From our morphological analyses during necrosis and nepoptosis, we identified changes in nucleus morphology and NE topography, using CNT/AFM probes at a nanometer scale, in DOX- and ETO-treated cells, at 72 h post-treatment. Based on the analysis of AFM images, we determined that the nucleus area of HK-2 and DMSO-treated cells was 140.0 ± 20.3 μm^2^, and the volume was 18.7 ± 2.9 μm^3^ for 10 evaluated nuclei. DOX-treatment induced nucleus swelling, with a mean nucleus area of 492.1 ± 54.5 μm^2^ and volume of 87.6 ± 18.0 μm^3^, and the cells typically exhibited nuclei with a round shape and a distinct nuclear boundary. The nuclei of ETO-treated cells were also shown to undergo nuclear swelling, with a nucleus area of 436.1 ± 67.0 μm^2^ and a volume of 88.8 ± 20.7 μm^3^, and nuclei has a visibly irregular shape with indistinct nuclear boundaries (Fig. 4A,C,D and Supplementary Fig. S5). ETO-treatment caused significant NE rupturing, with the size of ruptures showing a mean height of 94.2 ± 28.1 nm and width of 644.3 ± 189.0 nm, for 20 evaluated ruptures. A large number of linked-fiber chromatin was released from nuclei, and each single chromatin exhibited a diameter of 70–100 nm (Fig. 4B). Additionally, the NE changes in the ETO-treated HK-2 cells were analyzed after 24 h, using CNT/AFM probes. Interestingly, the results showed characteristic features of early apoptotic cell death, including NE ruptures and DNA release (Supplementary Fig. S6). However, the changes observed for 24 h ETO-treated cells were less significant than those reported in cells treated for 72 h. Therefore, in the early apoptotic cell death stage, small NE ruptures and release of small amounts of DNA were reported, while in late apoptotic cell death stages, severe NE ruptures as well as an excessive release of DNAs were observed.

### Cell adhesion disruption and NE rupturing are induced by caspase during nepoptosis

**Figure 5:**
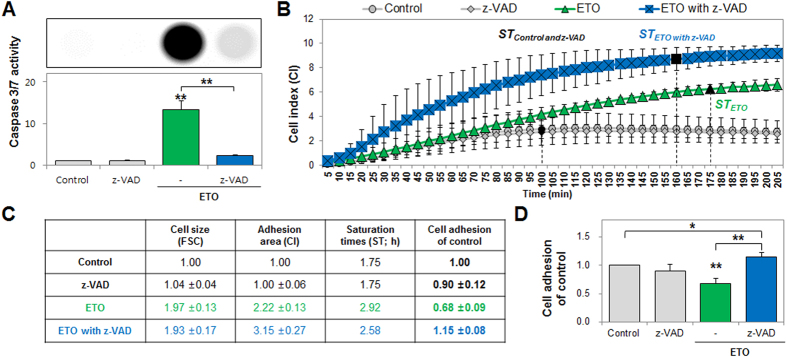
Inhibition of pan-caspase regained etoposide (ETO)-induced loss of cell adhesion. (**A**) Caspase 3/7 activity was measured using the Caspase-Glo 3/7 Assay and detected with a luminescence reader in ETO-treated cells co-treated with z-VAD, and the intensity is shown in the image. (**B**) Cells treated with ETO (green color) and co-treated with z-VAD (caspase inhibitor, blue color) were harvested at 72 h and cell adhesion, including cell index (CI) and saturation times (ST), were measured after detection by the xCELLigence system for 10 s intervals and monitored for 205 min. Black closed diagram indicate saturation (±1%) times. (**C**,**D**) The table shows cell size (FSC), adhesion area (CI), and saturation times (ST; h) at which cell adhesion was examined using computational analysis when compared to the control. The histogram shows cell adhesion levels using the derived formula for calculations (statistical analysis P-value of *P < 0.05, **P < 0.01) compared to the control or ETO.

**Figure 6:**
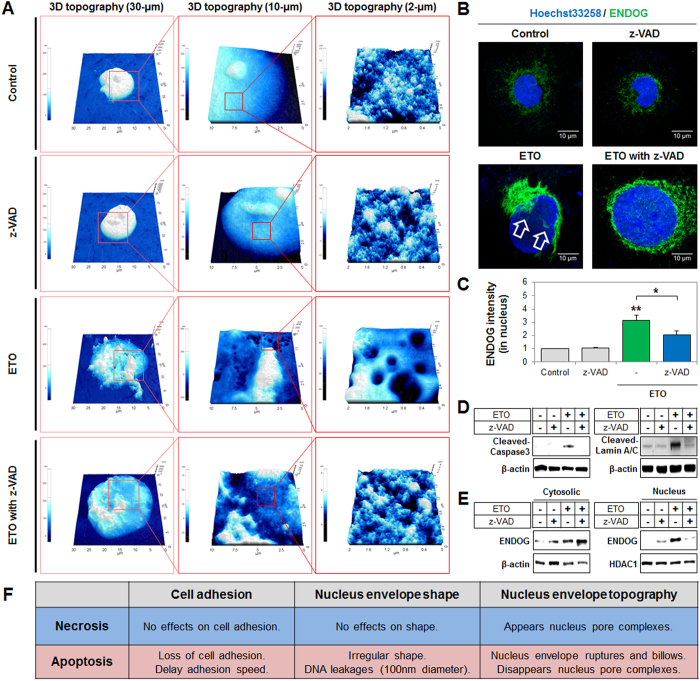
Inhibition of pan-caspase suppressed nuclear envelope rupturing and destruction of the nuclear pore complex, and regained endonuclease G translocation in the nuclei by etoposide (ETO). (**A**) Cells treated with ETO and cells co-treated with z-VAD were harvested. Nuclear extracts were seeded on culture dishes for 15 min and the nuclei were fixed. We measured nuclear envelope topography using a CNT/AFM probes system. Images shown are representative of 3D topography at 30-, 10-, and 2-μm scales. See also Supplementary Fig. S7. (**B**) Cells treated with ETO and co-treated with z-VAD regulated endonuclease G (green color) translocation levels, indicated by immunofluorescence staining and Hoechst 33258 (blue color) that was used for nuclear staining and were measured using confocal microscopy (scale bar represents 10 μm) at 72 h. Arrow indicates nuclear envelope ruptures (collapse Hoechst 33258 intensity) and/or endonuclease G translocation which also is also shown in Supplementary Fig. S8. (**C**) Translocation endonuclease G levels stained by immunofluorescence and Hoechst 33258 for nuclei that were measured using the Cellomics ArrayScan HCS Reader for at least 200 cells. Endonuclease G intensity was dependent on Hoechst 33258-positive area that is shown in the histogram compared to the control (statistical analysis P-value of *P < 0.05, **P < 0.01). (**D**) Cells treated with ETO and co-treated with z-VAD regulated expression of cleaved-caspase 3 and cleaved-lamin A/C protein in whole-extracted proteins evaluated using western blot analysis with β-actin as the loading control. (**E**) Cells treated with ETO and co-treated with z-VAD regulated expression of endonuclease G in the cytoplasm and nuclei as determined by western blot analysis with β-actin and HDAC1 as cytoplasm and nucleus loading controls, respectively. (**F**) The summarized cell adhesion, nuclear envelope shape, and nuclear envelope topography observed during necrosis and apoptosis.

Nepoptotic cell death generated cell adhesion disruption and NE rupturing, while necrotic cell death did not have any effect on cell adhesion or NE topography. Based on these results, we predicted, from a number of previous studies, that these outcomes were likely attributable to caspase activation during apoptosis^15^,^25^. Moreover, we demonstrated that caspases regulate changes in cell adhesion and NE topography using the pharmacological pan-caspase inhibitor, z-VAD, which almost completely inhibited ETO-induced caspase-3/7 activity (83.3% inhibition) at 72 h post-treatment (Fig. 5A). In these conditions, we demonstrated an alteration in cell adhesion using the xCELLigence system, and showed that inhibition of pan-caspase increased the CI from 2.2 ± 0.1-fold to 3.2 ± 0.3-fold and accelerated the ST from 175 min to 160 min when compared to ETO-treatment (Fig. 5B,C). In addition, we determined that cell nuclei of HK-2 and z-VAD-treated cells were round in shape with a distinct boundary, and also exhibited a large number of NPCs which were observed by using CNT/AFM probes (Fig. 6A and Supplementary Fig. S7B). Other studies showed similar results to ours^34^,^35^. The nuclei of ETO-treated cells clearly exhibited NE ruptures and DNA leakages, which were significantly suppressed by the inhibition of pan-caspase (Fig. 6A and Supplementary Fig. S7A). However, the inhibition of pan-caspase did not significantly alter nucleus area or volume of ETO-treated cells, based on 10 evaluated nuclei (Supplementary Fig. S7C).

### ENDOG is translocated in the nucleus through caspase-induced NE rupturing during nepoptosis

Translocation of endonuclease G (ENDOG) to the nucleus has been shown to trigger DNA fragmentation in the caspase-independent apoptosis pathway^36^; however, the mechanism for translocation is still unknown. Based on our results, we hypothesized that ENDOG translocation is mediated by caspase-induced NE rupturing, during ETO-induced nepoptosis. Thus, we measured translocation of ENDOG levels in pan-caspase inhibited cells. We confirmed the translocation of ENDOG (green color) to the nucleus of ETO-treated cells (indicated by the arrow) through disruption of NE using a nucleus-targeting dye (Hoechst 33258, blue color) and confocal microscopy (Fig. 6B, Supplementary Fig. S8A,B). In the pan-caspase-inhibited nuclei, ENDOG translocation and NE disruption were not apparent, as there were no significant changes in nucleus size from that of the uninhibited cells (Fig. 6B and Supplementary Fig. S8C). Similarly, results from the Cellomics ArrayScan HCS reader analysis showed that ETO-treatment increased ENDOG translocation in nuclei, by 3.1-fold from the control levels. Inhibition of pan-caspase resulted in a decrease in ENDOG translocation (33.7%), from levels of translocation for the ETO control cells, but did not significantly change the nucleus area- measured for at least 200 cells per well (Fig. 6C and Supplementary Fig. S7C). Furthermore, ETO treatment induced the expression of cleaved-caspase-3 and cleaved-lamin A/C (NE protein). However, the expression of these proteins and the translocation of ENDOG from cytosol to nucleus were significantly suppressed by the inhibition of pan-caspase (Fig. 6D,E).

## Discussion

The main purpose of the present study was to characterize cell adhesion, using xCELLigence analysis and NE topography obtained by AFM analysis, in necrotic and apoptotic cell death (Fig. 6F).

Cell adhesion plays an important role in cell migration, growth, differentiation, and morphology^9^, thus, it is associated with various human diseases, such as ischemia, asthma, diabetes, bacterial infections, and others^37^,^38^. In particular, the alteration of cell adhesion molecules has been shown to modulate invasion, metastasis and morphology in multistage carcinogenesis^39^. Currently, various cell adhesion-targeting drugs are being tested in clinical trials, in order to prevent the occurrence of carcinogenesis^40^,^41^,^42^. Amongst them, VS-6063 (formerly PF-04554878), has been reported to increase the apoptosis induced by paclitaxel, in ovarian cancer cells resistant to taxane, through the inhibition of the FAK-mediated chemoresistance signaling pathway^43^. This favored the application of these drugs in multiple clinical trials, such as trials for solid cancers, mesothelioma cancer, and ovarian cancer. In studies addressing cell death, it has been reported that apoptosis involved the disruption of key proteins for focal adhesion, including FAK and CAS, and of cytoskeletal proteins, including α-tubulin and phalloidin important for cell behavior and morphology, by the activation of caspases^13^,^16^. Generally, cell adhesion ability is measured by fibronectin and extracellular matrix protein-coated fluorometric or colorimetric detection assay kits, or by the enzyme-linked immunosorbent assay (ELISA), in studies of cancer and cell death^44^,^45^,^46^. However, these methods are limited by the indirect detection of cell adhesion ability and their detection only of the end-points. Here, we suggested the use of the xCELLigence system to directly measure and characterize the changes in cell adhesion during apoptosis and necrosis, in real-time. The xCELLigence system is used for cell-based measurements of cell viability, invasion, and migration, in real-time, which could not be detected by fluorometric or colorimetric methods^31^,^32^. Recently, some researchers used the xCELLigence system to study the migration of venous endothelial cells in chronic venous disease patients, or to study human neuronal cell networks^47^,^48^. In this study, we demonstrated, using xCELLigence system, that necrotic cell death generated cell swelling, while apoptotic cell death rapidly triggered loss of cell adhesion, and a decrease in cell-adhesion speed. The inhibition of pan-caspases limited destruction of the cell adhesion area during apoptosis to a level similar to that usually observed in necrosis. Overall, we determined that cell adhesion could be characterized in apoptosis and necrosis, based on the following properties: apoptosis induces loss of cell adhesion, through caspase involvement, but necrosis does not have any significant effect on cell adhesion. The calculation of a cell adhesion score presents a key indicator for both necrosis and apoptosis. During necrosis, an increase in the cell adhesion score is observed, while this score decreases during apoptosis. The xCELLigence method presented here, might represent a powerful tool for the measurement of cell adhesion, as it provides information regarding cell adhesion ability during necrosis and apoptosis, which serves to better characterize cell death. This method might also be a valuable aid to studies that address cell behavior in a variety of human diseases. In addition, the novel measurement ability realized by this method, and its concepts, will help in the design and production of new biosensors.

The NE separates the genetic information within a cell, from the cytoplasm, and controls the bi-directional transport of diverse molecules between the nucleus and cytoplasm. Moreover, changes in the NE have been reported to be associated with the development of multistage disease and carcinogenesis^49^,^50^. For example, both basal and squamous carcinoma cells present increased lamin A/C protein expression levels^51^. The NPC, composed of NUP88, has been found to be strongly expressed in multiple tumor cells, including adenocarcinoma cells, cervical carcinoma cells, and breast cancer cells^52^. In a study of stem cells, mouse and human ESCs both expressed B-type lamin proteins, but did not express A/C-type lamin proteins. However, lamin A/C proteins have been found to be expressed during the differentiation of both mouse and human ESCs^53^. Moreover, lamin B proteins are required for proper organogenesis, but do not have any effect on mouse ESCs^54^. Structural analysis of ESCs showed the presence of irregular and wide-shaped NEs in the intermembrane space of ESCs, in comparison to differentiated cells, through use of fluorescence staining and electron microscopy^55^. Further, apoptotic cell death showed disassembling of NE proteins, and structural changes in nucleus, such as shrinkage, NE rupture, irregular nucleus shape, and DNA leakage, which were measured by fluorescence staining and electron microscopy^29^,^56^,^57^. However, the study of structural and topographical changes of the NE using morphometric analysis is not ideal, due to the indirect detection of change by current methods, and the detection of only partial changes. Furthermore, necrosis-regulated NE alteration is not fully understood, and the methods for measuring these changes are limited. Here, we used a direct method of measurement, and determined that the nuclei of cells undergoing necrotic cell death exhibited a distinct rounded shape and clear nuclear boundary; NPCs were present in the NE, with a NE topography similar to that usually observed in normal nuclei. On the other hand, the activation of caspase triggered the formation of irregular-shaped nuclei and the disappearance of NPCs, in addition to the formation of significant ruptures and swelling in the NE, during apoptotic cell death. Therefore, this measurement method using CNT/AFM probes is a powerful tool, as it provides information regarding topographical alterations of the plasma membrane and the NE during necrosis and apoptosis, leading to better characterization of cell death features. Moreover, this method can be used for structural and functional analyses of the NE in studies of stem cells, and other cells in human multistage diseases.

In the mitochondrial or intrinsic signaling pathways, during apoptosis, the expression of BCL-2-associated X protein (BAX) or the BCL-2 antagonist/killer protein (BAK) cause the permeabilization of the mitochondrial outer membrane, and then production of apoptosis-inducing proteins, including cytochrome c (CYTC), caspase-activated DNase (CAD), apoptosis inducing factor (AIF), and ENDOG, which are released from the mitochondria to the cytoplasm^4^,^58^. CYTC forms an apoptosome by complex formation with apoptotic protease activating factor 1 (APAF1), and brings about DNA fragmentation and diverse protein destruction, through the activation of caspases^58^,^59^,^60^. DNA fragmentation is induced by nucleases, according to one of two mechanisms: caspase-dependent activation of CAD or caspase-independent release of AIF and ENDOG proteins from the mitochondria^1^,^4^,^61^. CAD released from the mitochondria is translocated into the nucleus, leading to DNA fragmentation, through the activation of caspase-3 in nucleus^62^. On the other hand, AIF and ENDOG are caspases that independently induce DNA fragmentation by translocating from the nucleus to the cytoplasm during apoptosis^36^,^63^. However, the mechanism for caspase and nuclease translocation in the nucleus is fully understood. In our study, we showed that apoptotic cell death was induced by ENDOG translocation and the leakage of DNA in the nucleus, through caspase-triggered NE ruptures. We also showed that caspases induced NE ruptures, and ultimately caused fragmentation of DNA and nuclear proteins, and leakage of DNA, in early-stage apoptosis. Translocation of caspases induced intermediate filament protein disassembly, triggering collapse of the NPCs and other proteins, by causing membrane infirmness in the NE. The progression of serious intermediate filament damage was triggered by pyknosis and/or karyorrhexis, through the collapse of structures due to external pressures during late-stage apoptosis.

The swelling of ETO-treated cells gradually caused apoptotic morphological changes, including cell shrinkage and apoptotic bodies (Supplementary Fig. S1 and Movie S1). On the other hand, necrotic cells stopped proliferating and maintained cell swelling when they were re-seeded in fresh media, but apoptosis continued to induce cell death and cell shrinkage. Hence, in DNA damage-induced cell death progressing from apoptosis to necrosis, as well as apoptosis alone, cells could not recover from the damage caused by destruction of the nucleus with DNA fragmentation. Taken together, our studies provide the structural and functional analyses of cell adhesion and nuclear envelope nano-topography in cell death.

## Methods

### Cell culture and treatment

HK-2 cells were purchased from American Type Culture Collection (ATCC, Manassas, VA, USA) and grown in RPMI 1640 media containing 10% fetal bovine serum (FBS) and 1% penicillin/streptomycin (Thermo Fisher Scientific Inc., Waltham, MA, USA) in an incubator system (humidified atmosphere of 5% CO_2_ at 37 °C; Thermo Fisher Scientific Inc.). Cells were treated with doxorubicin (DOX; 1 μM; Sigma-Aldrich Co. LLC, St. Louis, MO, USA), etoposide (ETO; 50 μM; Sigma-Aldrich Co. LLC) and z-VAD (25 μM; Santa Cruz Biotechnology, Inc., Dallas, TX, USA). Treated and untreated HK-2 cells were harvested using trypsin-EDTA (Thermo Fisher Scientific Inc.) for 3 min in a humidified atmosphere with 5% CO_2_ at 37 °C.

### Analysis of cell swelling measured using hemocytometer and FACS systems

HK-2 cells (4 × 10^5^ cells/6-cm culture dish) were grown overnight and treated with DOX and ETO for the specified times. After treatment, cells were collected and washed with 3 mL of phosphate buffered saline (PBS) and centrifuged at 200 × *g* for 5 min. For measurement, the harvested cells were first seeded in a hemocytometer (Paul Marienfeld GmbH & Co., KG, Bad Mergentheim, Germany); cell swelling was measured by phase contrast microscopy (E-scope i304, Macrotech Corporation, Goyang, South Korea) and analyzed using Scopephoto software. Next, forward scattered light (FSC) units indicating cell size were measured using FACS Aria III with Diva software (BD Biosciences., San Diego, CA, USA) for at least 1 × 10^5^ cells.

### Analysis of cell adhesion alteration using an xCELLigence system for real-time measurements

HK-2 cells (4 × 10^5^ cells/6-cm culture dish) were grown overnight and treated at the specified conditions for 72 h. Cells were then collected, washed with PBS (3 mL), and centrifuged at 200 × *g* for 5 min. The xCELLigence system (RTCA DP Analyzer, Roche Applied Science, Penzberg, Germany) was used for cell analysis. The system consisted of RTCA Resistor Plate 16 devices (ACEA Biosciences Inc., San Diego, CA, USA) placed in an incubator system (humidified atmosphere 5% CO_2_ at 37 °C; Thermo Fisher Scientific Inc.) and RTCA DP software (version 1.2) that was used to verify system conditions and for sample monitoring. Wells of the E-Plate 16 devices (ACEA Biosciences Inc.) were filled with 100 μL of RPMI 1640 media (containing 10% FBS and 1% penicillin/streptomycin), and the background value was measured using the RTCA DP software. Subsequently, harvested cells were counted using a hemocytometer (Paul Marienfeld GmbH & Co.), and cells (1 × 10^5^ cells/well in 100 μL RPMI 1640 media) were seeded on E-Plate 16 devices connected to the xCELLigence system. Real-time cell swelling and adhesion were measured using the RTCA DP software at 10 s intervals and monitored for 3 h. The real-time cell index was determined using the RTCA DP software, and a histogram representative of the cell index for 3 h was generated.

### Analysis of F-actin expression using confocal microscopy

Cells (4 × 10^5^ cells/6-cm culture dish) were grown overnight and treated at the specified conditions. Samples were then collected, washed with PBS (3 mL), and centrifuged at 200 × *g* for 5 min. Harvested cells were counted using a hemocytometer (Paul Marienfeld GmbH & Co.), seeded (1 × 10^4^ cells in RPMI 1640 media) in 6-cm culture dishes (SPL Life Sciences), and incubated under 5% CO_2_ at 37 °C (Thermo Fisher Scientific Inc.) for 3 h. After treatment, cells were fixed with 3.7% formaldehyde for 15 min and permeabilized by 0.2% Triton X-100 for 15 min. Subsequently, cells were blocked with 5.0% FBS for 1 h and incubated for 1 h with rhodamine-conjugated F-actin antibody (1:500; Invitrogen, Carlsbad, CA, USA). Hoechst 33258 reagent (5 μM; Sigma-Aldrich Co. LLC) for nuclear staining was added to the samples for 30 min at room temperature, and samples were washed with PBS for 3 times. F-actin fluorescence intensity and cell adhesion area were measured using confocal microscopy to capture images of stained cells (LSM-700; Carl Zeiss MicroImaging GmbH, Jena, Germany), and the images were analyzed with Zen 2009 software.

### Analysis of cell adhesion and cell swelling using phase contrast microscopy

HK-2 **c**ells (4 × 10^5^ cells/6-cm culture dish) were grown overnight and then treated with DOX and ETO for 72 h, after which cells were collected, washed with PBS (3 mL), and centrifuged at 200 × *g* for 5 min. Harvested cells were seeded in culture dishes in an incubator system (humidified atmosphere of 5% CO_2_ at 37 °C) for 3 h, and cell swelling was measured by phase contrast microscopy (E-scope i304, Macrotech Corporation), and then analyzed using the Scopephoto software.

### Analysis of plasma membrane topography measured using a CNT/AFM probes system

Cells (4 × 10^5^ cells/6-cm culture dish) were grown overnight and treated at the specified conditions. Samples were then collected, washed with PBS (3 mL), and centrifuged at 200 × *g* for 5 min. Harvested cells were counted using a hemocytometer (Paul Marienfeld GmbH & Co.) and then seeded (1 × 10^4^ cells in RPMI 1640 media) in 6-cm culture dishes (SPL Life Sciences) that were incubated in 5% CO_2_ at 37 °C (Thermo Fisher Scientific Inc.) for 3 h. Subsequently, samples were fixed with 3.7% formaldehyde for 15 min and then washed with PBS and deionized water 3 times. Atomic force microscopy (AFM) images were obtained in a non-contact mode with an XE-100 AFM system (Park Systems Corp., Suwon, Korea). Carbon nanotubes (CNTs) attached to the AFM cantilevers with spring constants of 42 N/m were used with a resonance frequency of 310 kHz. AFM image analyses, consisting of 3D topography and enhanced color topography, were performed using XEI software (Park Systems Corp.).

### Nuclear extraction

Cells (4 × 10^5^ cells/6-cm culture dish) were grown overnight and then treated at the specified conditions for 72 h. Cells were then collected, washed with PBS (3 mL), and centrifuged at 200 × *g* for 5 min. Nuclear extraction was performed using NE-PER Nuclear and Cytoplasmic Extraction Reagents (Thermo Fisher Scientific Inc.). Cytoplasmic Extraction Reagent I (100 μL) containing protease and phosphatase inhibitor cocktails (1:100, Thermo Fisher Scientific Inc.) was added to harvested cells for 10 min on ice (4 °C) while tapping the samples. Cytoplasmic Extraction Reagent II (5.5 μL) was then added to the samples for 1 min while tapping the samples. Samples were centrifuged at 16,000 × *g* for 5 min at 4 °C and then kept on ice (4 °C).

### Analysis of nucleus swelling using a hemocytometer and staining with Hoechst 33258 and PI

Extracted nuclei were seeded on a hemocytometer (Paul Marienfeld GmbH & Co.) and measured using phase contrast microscopy (E-scope i304, Macrotech Corporation). Analysis was performed using Scopephoto software. Extracted nuclei were also seeded on 24-well plates with a coverslip insert (SPL Life Sciences., Pochun, Korea) for 15 min at room temperature and then washed at least 3 times with PBS. After the nuclei were fixed with 3.7% formaldehyde for 15 min, they were stained with Hoechst 33258 (5 μM, 1:1000, Sigma-Aldrich Co. LLC) and propidium iodide (PI; 5 μM, 1:1000, Sigma-Aldrich Co. LLC) dyes for 15 min. The nuclei were measured using confocal microscopy (LSM-700, Carl Zeiss MicroImaging GmbH) and analyzed using the Zen 2009 software.

### Analysis of nuclear envelope topography measured using a CNT/AFM probes system

A volume of 1 mL of PBS was added to the nuclear extracts, and samples were seeded on 6-cm culture dishes for 15 min and then washed at least 3 times with PBS. Subsequently, all samples were fixed with 3.7% formaldehyde for 15 min and then washed with PBS and deionized water 3 times. Atomic force microscopy (AFM) images were obtained in non-contact mode with an XE-100 AFM system (Park Systems Corp.). Carbon nanotubes (CNTs) attached to the AFM cantilevers with spring constants of 42 N/m were used with a resonance frequency of 310 kHz. AFM image analyses, consisting of 3D topography and measurements of area and volume, were performed using XEI software (Park Systems Corp.).

### Analysis of caspase 3/7 activity

Cells (4 × 10^5^ cells/6-cm culture dish) were grown overnight and then treated with ETO and/or z-VAD for 72 h. Samples were collected, washed with PBS (3 mL), and centrifuged at 200 × *g* for 5 min. Harvested cells were counted using a hemocytometer (Paul Marienfeld GmbH & Co.) and seeded (1 × 10^4^ cells/well) in 96-well plates (Greiner Bio-One., Frickenhausen, Germany). Caspase 3/7 activity was determined using a Caspase-Glo 3/7 Assay (Promega Corporation, Madison, WI, USA), according to the manufacturer’s instructions, and detected using a SPECTRAmax GEMINI fluorescence microplate reader (Molecular Devices Inc., Sunnyvale, CA, USA), with luminescence intensity detected using a Fuji LAS-3000 system (Fujifilm, Tokyo, Japan).

### Analysis of endonuclease G translocation in the nucleus using confocal microscopy and a Cellomics ArrayScan HCS Reader system

Cells (1 × 10^4^ cells/well) were seeded in a black 96-well *μ*CLEAR-plate (Greiner Bio-One), grown overnight, and then treated with ETO and/or z-VAD for 72 h. After treatment, cells were fixed with 3.7% formaldehyde for 15 min and permeabilized by 0.2% Triton X-100 for 15 min. Subsequently, cells were blocked with 5.0% FBS for 1 h and incubated for 1 h with primary endonuclease G (ENDOG) antibody (1:500; Santa Cruz Biotechnology, Santa Cruz, CA, USA), followed by treatment with a secondary specific antibody conjugated to AlexaFluor 488 (Invitrogen) for 1 h. Hoechst 33258 reagent (5 μM; Sigma-Aldrich Co. LLC) for nuclear staining was added to the samples for 30 min at room temperature, and samples were washed with PBS 3 times. Stained cells were measured using a Cellomics ArrayScan HCS Reader (20x objective lens, Thermo Fisher Scientific Inc.) for at least 200 cells in each well. ENDOG fluorescence intensity in the nuclei was analyzed using ArrayScan VTI (600 series) version 6.6.1.3 software. The plate was measured using confocal microscopy to capture images of stained cells (LSM-700, Carl Zeiss MicroImaging GmbH), and the images were analyzed with Zen 2009 software.

### Analysis of protein expression levels using western blotting

Cells (4 × 10^5^ cells/6-cm culture dish) were grown overnight and then treated with ETO and/or z-VAD for 72 h. Samples were collected, washed with PBS (3 mL), and centrifuged at 200 × *g* for 5 min. Whole-protein extracts were obtained using M-PER solution (Thermo Fisher Scientific Inc.), and nuclear and cytoplasm proteins were extracted using NE-PER Nuclear and Cytoplasmic Extraction Reagents (Thermo Fisher Scientific Inc.) according to the manufacturer’s instructions. Protein concentrations were measured using a BCA assay (Sigma-Aldrich Co. LLC) according to the manufacturer’s instructions. Protein samples (20–50 μg) were separated by molecular weight using 10–12% sodium dodecyl sulfate polyacrylamide gel electrophoresis (SDS-PAGE, Mini-PROTEAN Tetra Cell, Bio-Rad Laboratories, Hercules, CA, USA) system. Samples were subsequently transferred to nitrocellulose membranes (Hybond ECL; Amersham Pharmacia Biotech, Inc., Piscataway, NJ, USA) using a Mini Trans-Blot Electrophoretic Transfer Cell system (Bio-Rad Laboratories) containing transfer buffer at 70 V for 4 h at 4 °C. The membranes were blocked with 5% non-fat dried milk for 1 h on a shaker (Fine PCR, Seoul, Korea). The membranes were immuno-blotted using specific primary antibodies (1:500–1000): HDAC1, β-actin, cleaved-caspase 3, lamin A/C, and ENDOG (Santa Cruz Biotechnology) at 4 °C overnight. After incubation, the membranes were washed 5 times with PBST (containing 0.05% Tween-20 in PBS), and they were incubated with anti-mouse or anti-rabbit peroxidase-conjugated antibodies (1:1000, Thermo Fisher Scientific Inc.) for 2 h. Protein levels were measured using SuperSignal West Pico ECL solution (Thermo Fisher Scientific Inc.) and detected using a Fuji LAS-3000 system (Fujifilm).

### Statistical analysis

All data represents values from at least 3 independent experiments. Statistical significance was defined by P-values of *P < 0.05 or **P < 0.01 using one-way analysis of variance (ANOVA) statistical analyses from the SigmaPlot software version 12.0 (Systat Software Inc., San Jose, CA, USA).

## Additional Information

**How to cite this article**: Kwon, H.-K. *et al.* Structural and functional analysis of cell adhesion and nuclear envelope nano-topography in cell death. *Sci. Rep.* **5**, 15623; doi: 10.1038/srep15623 (2015).

## Supplementary Material

Supplementary Information

Supplementary Movie S1
