# Supplementary material for: Structural and functional analysis of cell adhesion and nuclear envelope nano-topography in cell death
Source: Sci Rep. 2015 Oct 22;5:15623. doi: 10.1038/srep15623 (PMC4614995; doi:10.1038/srep15623)
Supplement: Supplementary Information [file srep15623-s1.pdf]

< *Supporting Information*>

Structural and functional analysis of cell adhesion and nuclear envelope nano-topography in cell death

Hyuk-Kwon Kwon[1,2], Jae-Hyeok Lee[1,2,3], Hyeon-Jun Shin[1], Jae-Ho Kim[1] & Sangdun Choi[1]

[1]Department of Molecular Science and Technology, Ajou University, Suwon 443-749, Korea
[3]Department of Materials Science and Engineering, Northwestern University, Evanston, Illinois 60208, USA

[2]These authors contributed equally to this work.

Corresponding author:
Sangdun Choi
Department of Molecular Science and Technology, Ajou University, Suwon 443-749, Korea
Phone: +82-31-219-2600
Fax: +82-31-219-1615
E-mail: sangdunchoi@ajou.ac.kr

Methods

Cell culture and treatment. HK-2 cells were purchased from American Type Culture Collection (ATCC, Manassas, VA, USA) and grown in RPMI 1640 media containing 10% fetal bovine serum (FBS) and 1% penicillin/streptomycin (Thermo Fisher Scientific Inc., Waltham, MA, USA) in an incubator system (humidified atmosphere of 5% CO_2 at 37°C; Thermo Fisher Scientific Inc.). Cells were treated with doxorubicin (DOX; 1 μM; Sigma-Aldrich Co. LLC, St. Louis, MO, USA), etoposide (ETO; 50 μM; Sigma-Aldrich Co. LLC) and z-VAD (25 μM; Santa Cruz Biotechnology, Inc., Dallas, TX, USA). Treated and untreated HK-2 cells were harvested using trypsin-EDTA (Thermo Fisher Scientific Inc.) for 3 min in a humidified atmosphere with 5% CO_2 at 37°C.

Analysis of cell viability measured using MTT assay. Cells (5×10^3 cells/well) were seeded on 96-well plates (BD Biosciences.) and grown overnight. Cells were then treated with doxorubicin (DOX) and etoposide (ETO) for 24, 48, and 72 h. Cell viability was determined using a colorimetric 1-(4,5-Dimethylthiazol-2-yl)-3,5-diphenylformazan (MTT) solution (Sigma-Aldrich Co. LLC). The MTT solution was added (100 μL/well) to the wells, and the samples were incubated at 37°C for 3 h. The MTT solution was removed, and DMSO solution (100 μL/well; Sigma-Aldrich Co. LLC) was added for 30 min. Cell viability was measured using a microplate spectrophotometer system (Molecular Devices Inc.) at 540 nm.

Analysis of apoptosis and necrosis using annexin V/PI double staining. Cells (4×10^5 cells/6-cm culture dish) were grown overnight and then treated with DOX and ETO for 24, 48, and 72 h. Samples were collected and washed with PBS (3 mL) and centrifuged at $200 \times g$ for 5 min. Harvested cells were added to cold binding buffer (100 μL) containing Annexin V (10 μL) and propidium iodide (PI; 10 μL) dyes (BD Biosciences.) in a brown tube that was allowed to react for 15 min at room temperature. After this incubation, cold binding buffer (200 μL) was added and samples were maintained at 4°C. Apoptosis and necrosis were analyzed using a FACS Aria™ III system with Diva™ software (BD Biosciences.).

Analysis of cell swelling and cell adhesion using a real-time live-cell imaging system. Cells (4×10^5 cells/6-cm dish) were grown overnight and then treated with DOX and ETO

for 72 h. Samples were collected, washed with PBS (3 mL), and centrifuged at 200 × *g* for 5 min. Harvested cells seeded were seeded (1 × 10^5 cells in 1.5 mL of RPMI 1640 media) on a Glass-Bottom Cell Culture Dish (Nest Biotechnology, Jiangsu, China), and measured using a real-time live-cell imaging system (Delta Vision RT, Applied Precision, Issaquah, WA, USA) combined with interval microscopy (Olympus, Tokyo, Japan) in an environmental control chamber (Weather Station, Precision Control LLC, http://www.precisioncontrol.net) with 5% CO_2 at 37°C. Images were captured using a 60× lens, dropping 1.42 numerical aperture oil immersions, and then detected using a CCD camera (CoolSNAPHQ Fast CCD camera, Roper Scientific, Duluth, GA, USA). Images were analyzed using softWoRxTM Imaging Workstation software (Applied Precision).

Analysis of cell adhesion and cell swelling using phase contrast microscopy. HK-2 **c**ells (4 × 10^5 cells/6-cm culture dish) were grown overnight and then treated with DOX and ETO for 72 h, after which cells were collected, washed with PBS (3 mL), and centrifuged at 200 × *g* for 5 min. Harvested cells were seeded in culture dishes in an incubator system (humidified atmosphere of 5% CO_2 at 37°C) for 3 h, and cell swelling was measured by phase contrast microscopy (E-scope i304, Macrotech Corporation), and then analyzed using the Scopephoto software.

Analysis of plasma membrane topography measured using a CNT/AFM probes system. Cells (4 × 10^5 cells/6-cm culture dish) were grown overnight and treated at the specified conditions. Samples were then collected, washed with PBS (3 mL), and centrifuged at 200 × *g* for 5 min. Harvested cells were counted using a hemocytometer (Paul Marienfeld GmbH & Co.) and then seeded (1 × 10^4 cells in RPMI 1640 media) in 6-cm culture dishes (SPL Life Sciences) followed by incubation in 5% CO_2 at 37°C (Thermo Fisher Scientific Inc.) for 3 h. Subsequently, samples were fixed with 3.7% formaldehyde for 15 min and then washed with PBS and deionized water 3 times. Atomic force microscopy (AFM) images were obtained in a non-contact mode with an XE-100 AFM system (Park Systems Corp., Suwon, Korea). Carbon nanotubes (CNTs) attached to the AFM cantilevers with spring constants of 42 N/m were used with a resonance frequency of 310 kHz. AFM image analyses, consisting of 3D topography, enhanced color topography, roughness and height, were performed using XEI software (Park Systems Corp.).

Analysis of nucleus swelling measured using a Cellomics ArrayScan HCS Reader and confocal microscopy system. Cells (1×10^4 cells/well) were seeded on a black, 96-well μCLEAR-Plate, (Greiner Bio-One.) and grown overnight. Cells were then treated with DOX and ETO for 72 h. Cells were fixed with 3.7% formaldehyde for 15 min and permeabilized by 0.2% Triton X-100 for 15 min. Subsequently, cells were blocked with 5.0% fetal bovine serum (FBS) for 1 h and washed with PBS 3 times. Samples were stained using Hoechst 33258 and PI (5 μM and 5 μM, respectively) for 30 min at room temperature and washed with PBS 3 times. Stained cells were measured using a Cellomics ArrayScan HCS Reader (20× objective lens, Thermo Fisher Scientific Inc.) for at least 200 cells in each well. Nucleus area and fluorescence intensity were analyzed using ArrayScan VTI (600 series) Version 6.6.1.3 software. The plate was measured using confocal microscopy to image stained cells (LSM-700, Carl Zeiss MicroImaging GmbH.), and the image were analyzed with Zen 2009 software.

Analysis of nuclear envelope topography measured using a CNT/AFM probes system. A volume of 1 mL of PBS was added to the nuclear extracts, and samples were seeded on 6-cm culture dishes for 15 min and then washed at least 3 times with PBS. Subsequently, all samples were fixed with 3.7% formaldehyde for 15 min and then washed with PBS and deionized water 3 times. Atomic force microscopy (AFM) images were obtained in non-contact mode with an XE-100 AFM system (Park Systems Corp.). Carbon nanotubes (CNTs) attached to the AFM cantilevers with spring constants of 42 N/m were used with a resonance frequency of 310 kHz. AFM image analyses, consisting of 3D topography and measurements of area and volume, were performed using XEI software (Park Systems Corp.).

Analysis of endonuclease G translocation in the nucleus and nucleus area measured using a Cellomics ArrayScan HCS Reader and confocal microscopy system. Cells (1×10^4 cells/well) were seeded on a black, 96-well μCLEAR-Plate (Greiner Bio-One.) and grown overnight. Cells were treated with ETO and/or z-VAD for 72 h. Following treatment, cells were fixed with 3.7% formaldehyde for 15 min and permeabilized by 0.2% Triton X-100 for 15 min. Subsequently, cells were blocked with 5.0% fetal bovine serum for 1 h and incubated for 1 h with primary endonuclease G (ENDOG) antibody (1:500; Santa Cruz Biotechnology)

and then treated with a secondary specific antibody conjugated to AlexaFluor 488 (Invitrogen) for 1 h. Hoechst 33258 reagent (5 μM; Sigma-Aldrich Co. LLC) for nuclear staining was added and samples were incubated for 30 min at room temperature. Cells were washed with PBS 3 times, and Hoechst33258 stained-cells were measured using a Cellomics ArrayScan HCS Reader (20× objective lens, Thermo Fisher Scientific Inc.) for at least 200 cells in each well. Nucleus area was analyzed using ArrayScan VTI (600 series) Version 6.6.1.3 software. The plate was measured using confocal microscopy to image stained cells (LSM-700, Carl Zeiss MicroImaging GmbH.), and the images were analyzed with Zen 2009 software.

Statistical analysis. All data represents values from at least 3 independent experiments. Statistical significance was defined by P-values of $^*P < 0.05$ or $^{**}P < 0.01$ using one-way analysis of variance (ANOVA) statistical analyses from the SigmaPlot software version 12.0 (Systat Software Inc., San Jose, CA, USA).

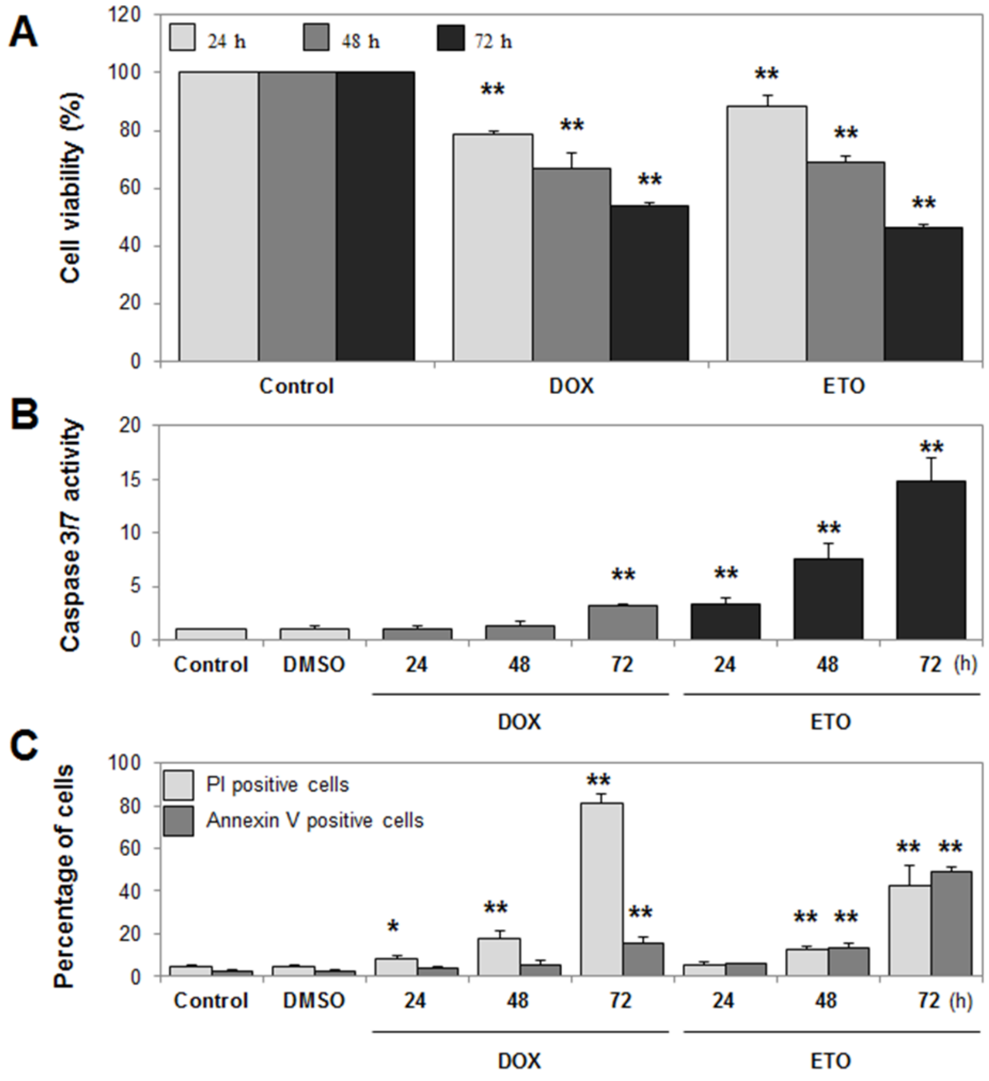

Supplementary Figure S1. Doxorubicin (DOX) and etoposide (ETO) induced caspase 3/7 activity and cell death in different manners. (A) DOX- (1 μM) and ETO- (50 μM) induced cytotoxicity measured using a cell viability analysis (MTT assay) at increasing times. (B) DOX and ETO increased caspase 3/7 activity as measured using a Caspase-Glo 3/7 Assay over increasing times. (C) DOX and ETO induced necrotic and/or apoptotic cell death, as indicated by Annexin V and PI double staining over increasing times. The histogram shows the percentage of Annexin V- (indicative of apoptosis) and PI- (indicative of necrosis) positive cells. All histograms indicate statistical analysis performed using P-value of $*P < 0.05$, $**P < 0.01$).

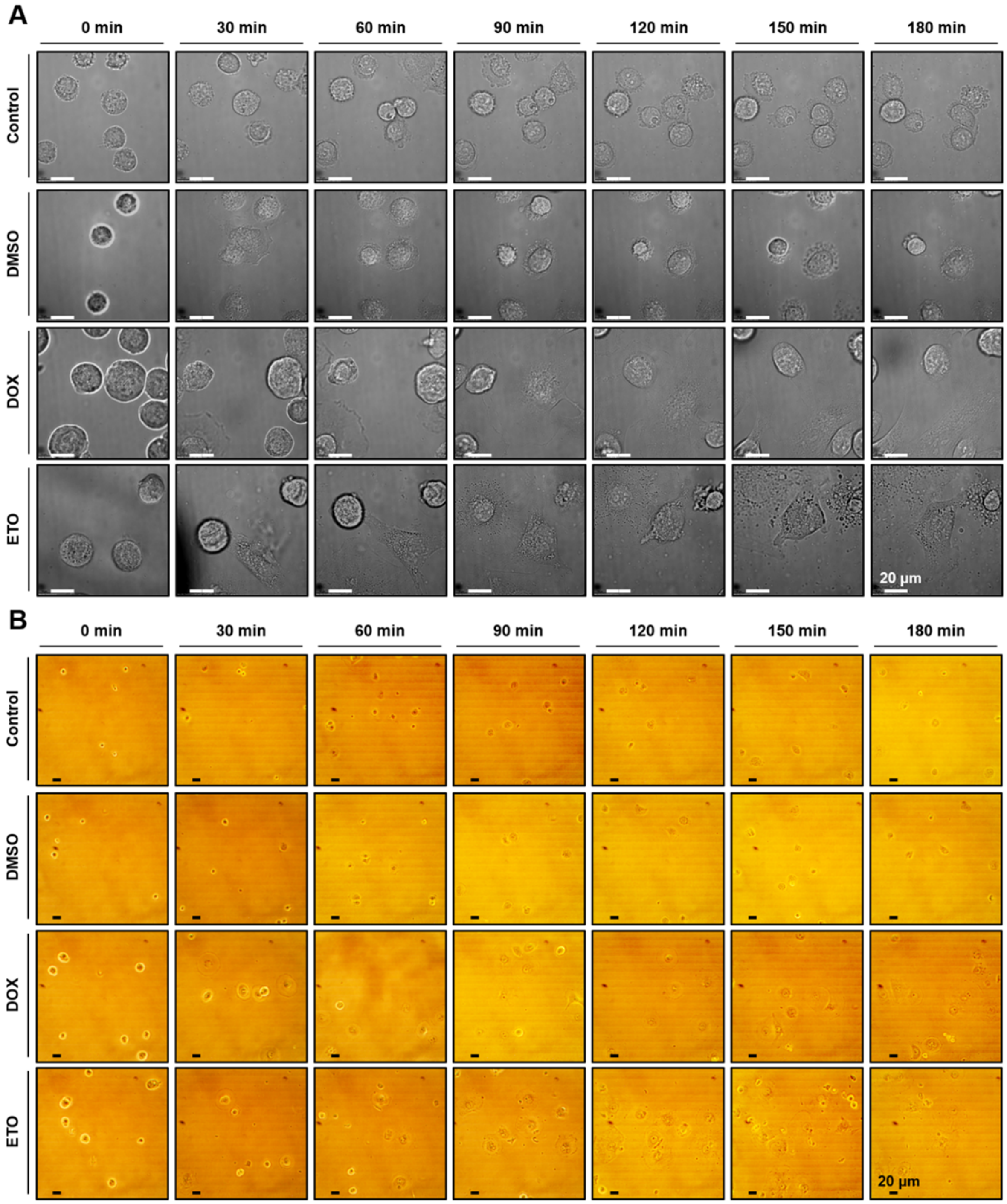

Supplementary Figure S2. Doxorubicin (DOX) and etoposide (ETO) induced cell swelling and expansion of cell adhesion area. DOX- and ETO-treated cells were seeded in culture dishes, and cell adhesion was measured using live-cell imaging microscopy at real-time (A) and phage contrast microscopy at 30 min time intervals (B). Images shown represent cell adhesion at the indicated times (scale bar represents 20 µm). A supplemental movie file is also provided (Supplemental Movie S1).

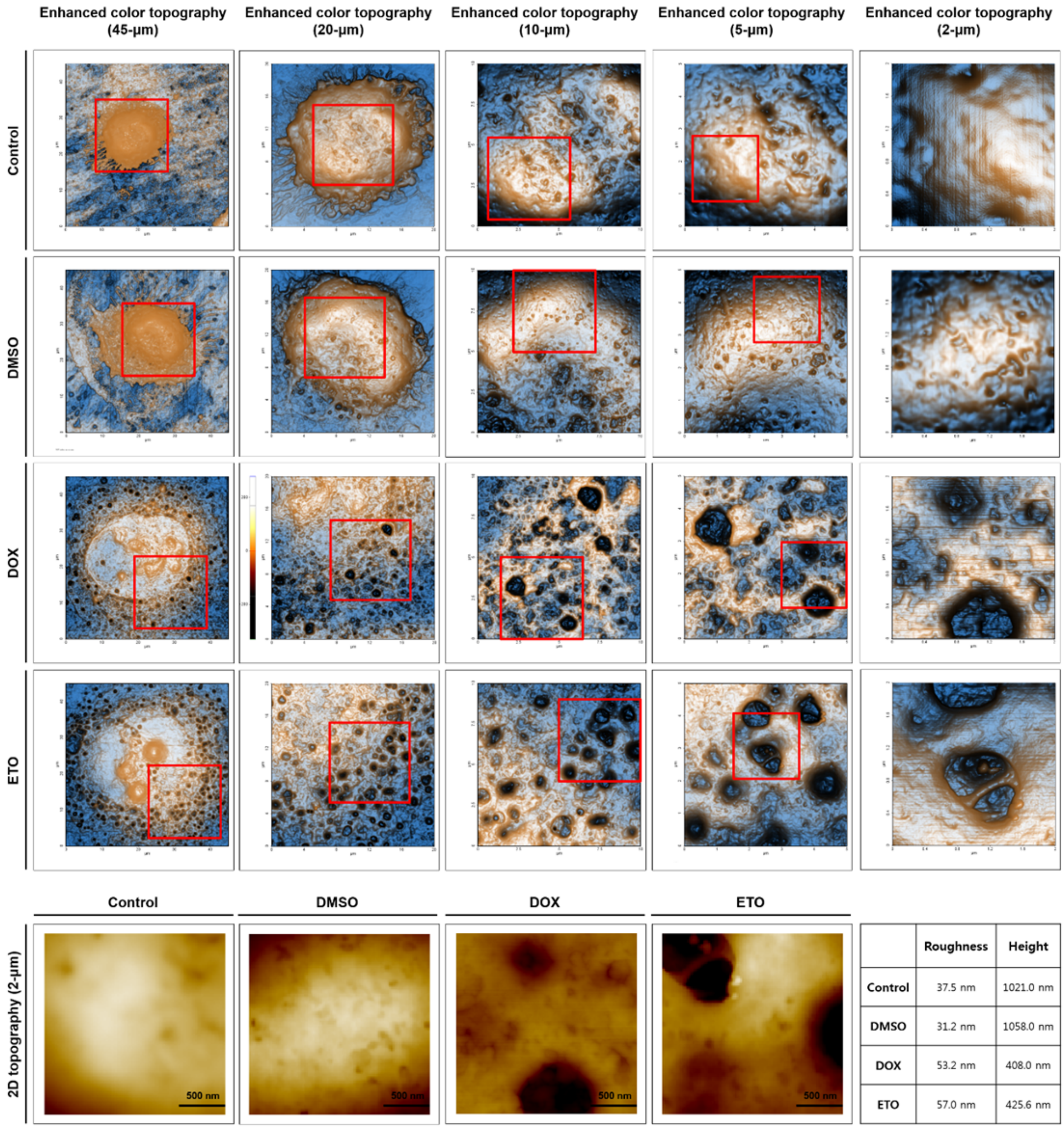

	Roughness	Height
Control	37.5 nm	1021.0 nm
DMSO	31.2 nm	1058.0 nm
DOX	53.2 nm	408.0 nm
ETO	57.0 nm	425.6 nm

Supplementary Figure S3. Doxorubicin (DOX) and etoposide (ETO) preserved necrotic morphological changes, including cell swelling and plasma membrane rupturing. DOX- and ETO-treated cells were harvested and seeded in culture dishes for 3 h, after which time topographic changes of the plasma membrane were measured using CNT/AFM probes. Images are shown with enhanced color topography at 2- to 45-µm scales, roughness at 2-µm scale and height at 45-µm scale using the XEI software.

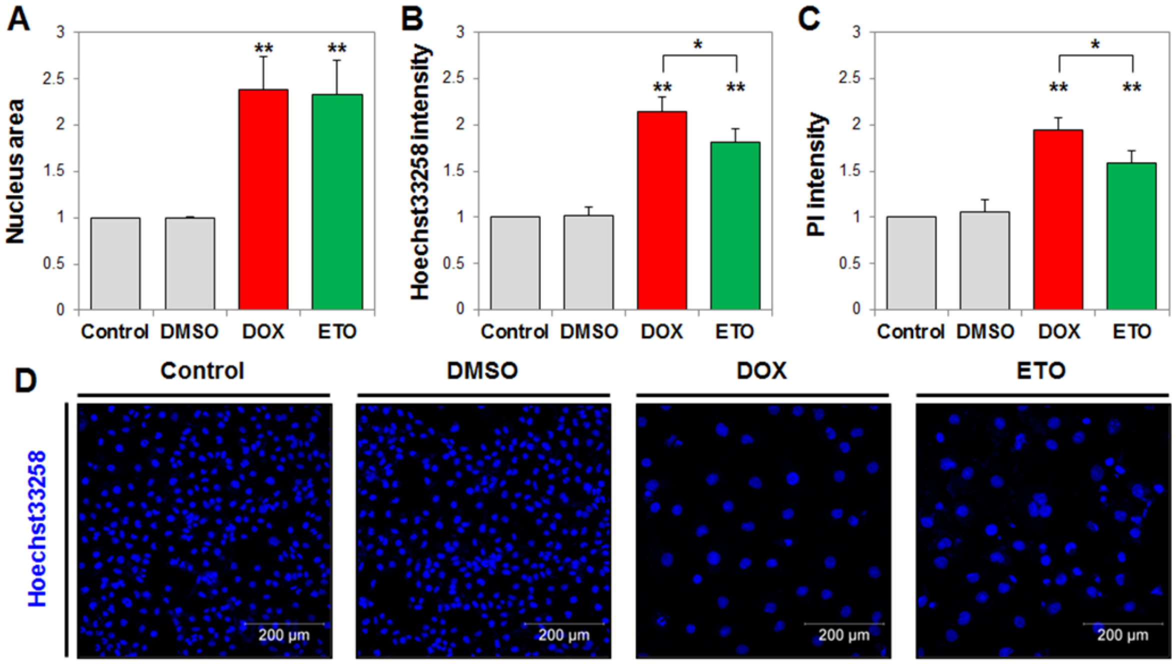

Supplementary Figure S4. Doxorubicin (DOX) and etoposide (ETO) treatment induced nuclear swelling. DOX and ETO affected nucleus area, as indicated by staining with Hoechst 33258 and PI nuclear-targeting dyes. Staining was then measured using a Cellomics ArrayScan HCS Reader for at least 200 cells. Nucleus area (A), Hoechst 33258 intensity (B), and PI intensity (C) were compared to the control (statistical analysis P-value of $*P < 0.05$, $**P < 0.01$). (D) Nucleus swelling measured using confocal microscopy to visualize Hoechst 33258 staining (scale bar represents 200 μm).

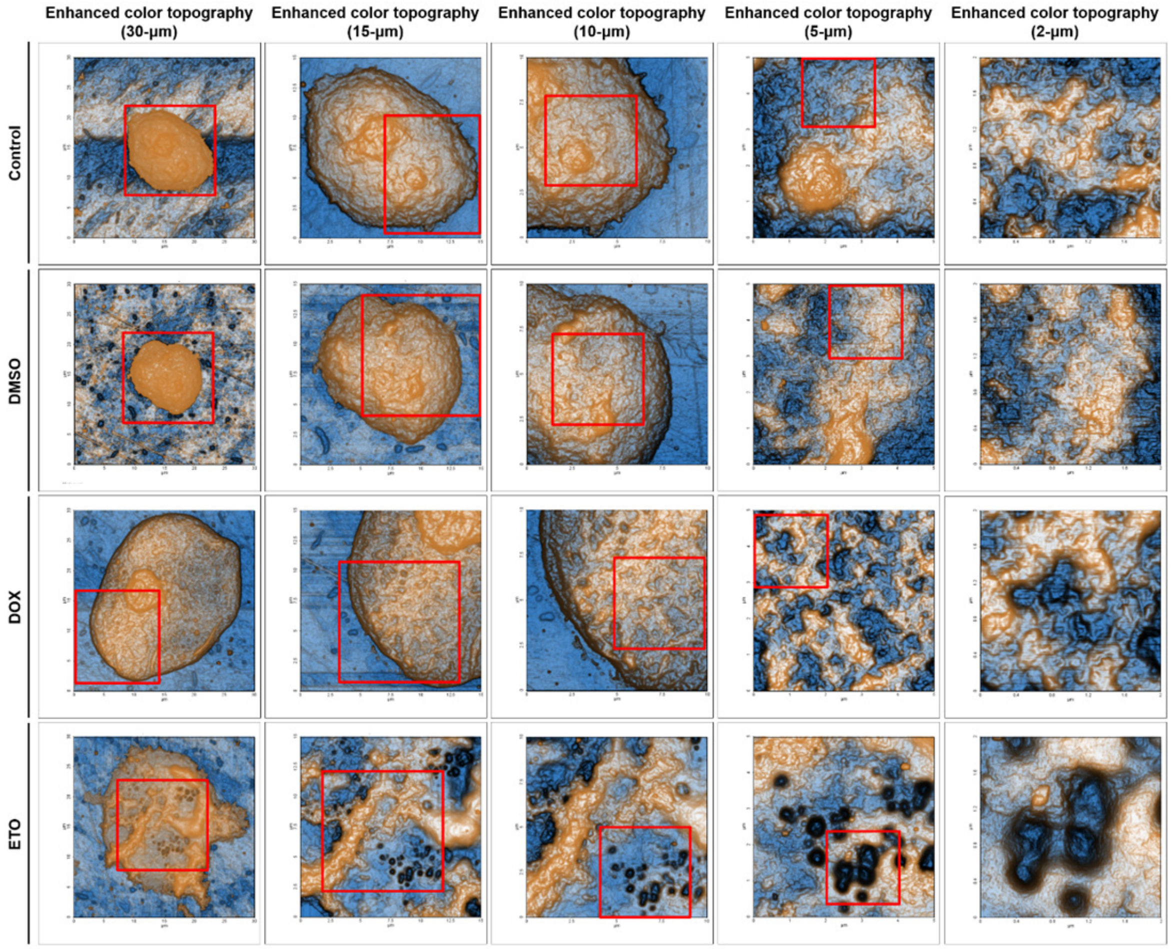

Supplementary Figure S5. Doxorubicin (DOX) and etoposide (ETO) generated nucleus swelling and varied nuclear envelope topography. Nuclear extracts from control cells and cells treated with DMSO, DOX, and ETO were seeded in culture dishes for 15 min and the nuclei were fixed. The nuclear envelope topography was measured using a CNT/AFM probe system. Images shown are enhanced color topography images generated by XEI software at 2- to 30-µm scales.

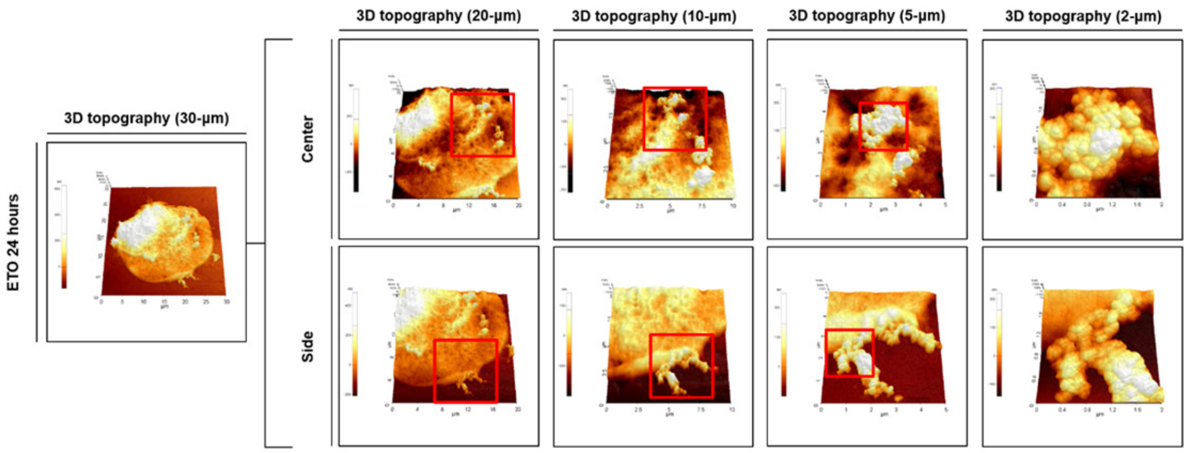

Supplementary Figure S6. Etoposide treatment induced nuclear envelope ruptures. Nuclear extracts from etoposide-treated cells were seeded in culture dishes for 15 min. The nuclear envelope topography was measured using a CNT/AFM probe system. 3D topography images were generated by XEI software at 2- to 30-μm scales.

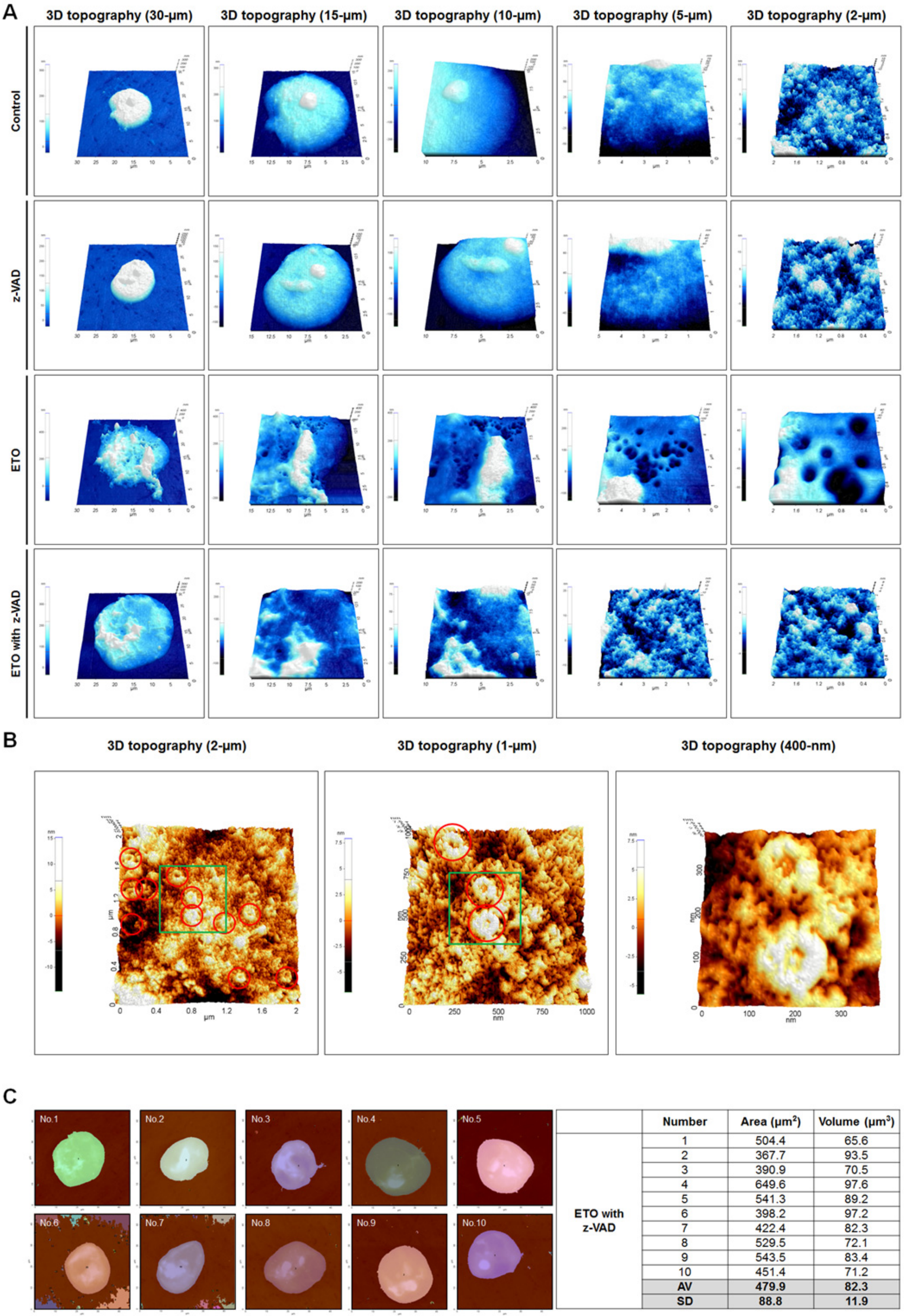

	Number	Area (μm²)	Volume (μm³)
ETO with z-VAD	1	504.4	65.6
	2	367.7	93.5
	3	390.9	70.5
	4	649.6	97.6
	5	541.3	89.2
	6	398.2	97.2
	7	422.4	82.3
	8	529.5	72.1
	9	543.5	83.4
	10	451.4	71.2
	AV	479.9	82.3
	SD	88.8	11.9

Supplementary Figure S7. Inhibition of pan-caspase suppressed etoposide-generated nuclear envelope rupture. (A) Cells treated with ETO and z-VAD for 72 h were harvested

and their nuclear extracts were seeded in culture dishes for 15 min. The nuclei were fixed and the nuclear envelope topography was measured using a CNT/AFM probe system. Images shown represent the 3D topography as determined using XEI software at 2- to 30-µm scales. (B) Images represent the 3D topography of nuclear pore complexes in control cells measured by XEI software at 2-µm, 1-µm, and 400-nm scales. (C) Nucleus area and volume were measured in cells treated with ETO and z-VAD using XEI software at 45-µm scale for 10 nuclei.

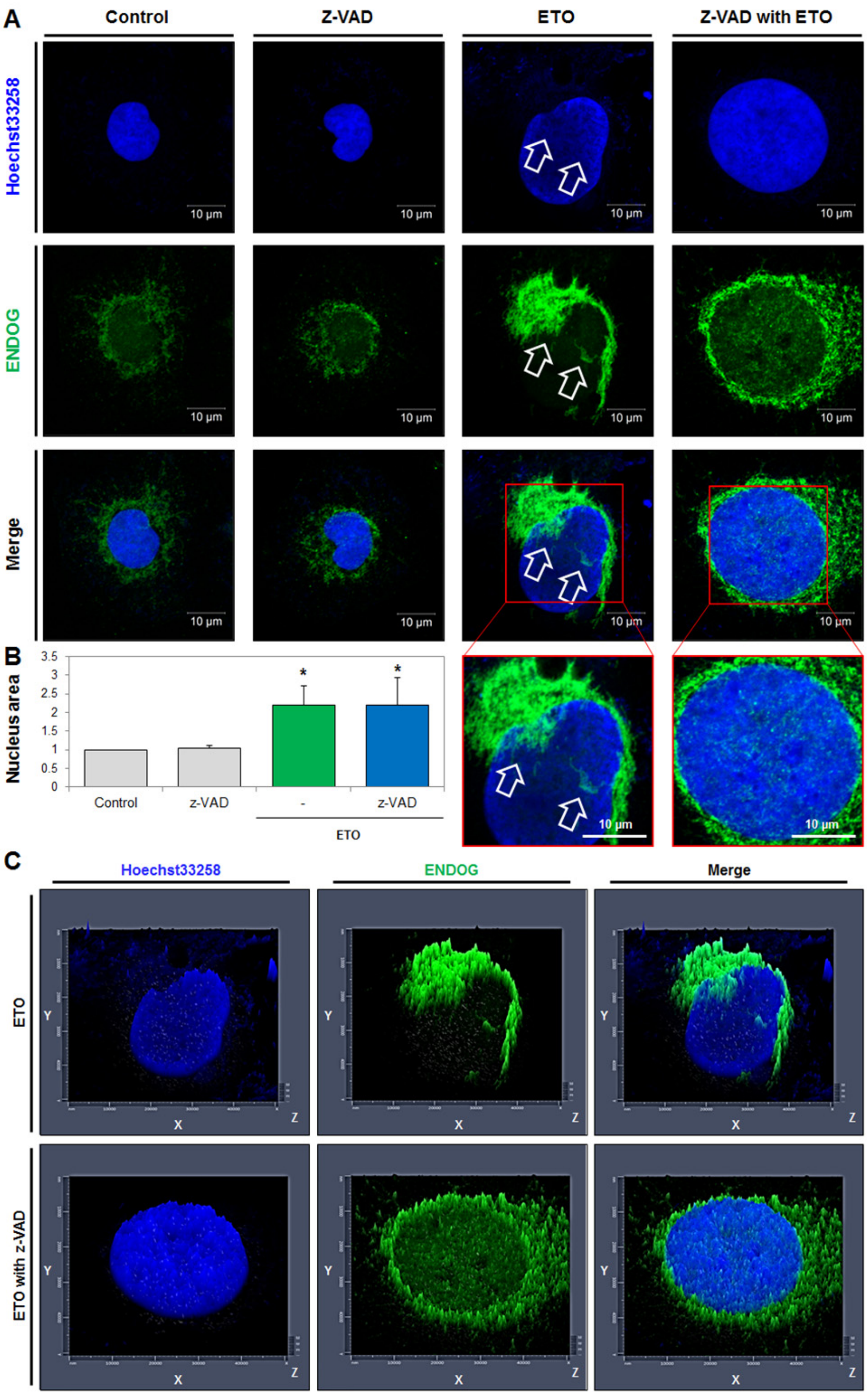

Supplementary Figure S8. Inhibition of pan-caspase suppressed etoposide (ETO)-induced endonuclease G translocation to the nucleus and did not affect nucleus area. (A)

Cells treated with ETO and co-treated with z-VAD regulated endonuclease G (green color) translocation levels indicated by immunofluorescence staining. Hoechst 33258 (blue color) was used for nuclear staining and was measured using confocal microscopy (scale bar represents 10 μm) at 72 h. The arrows indicate nuclear envelope ruptures and/or endonuclease G translocation. (B) Cells treated with ETO and z-VAD regulated nucleus area, as measured using the Cellomics ArrayScan HCS Reader for at least 200 cells stained with Hoechst 33258 (statistical analysis P-value of *$P < 0.05$, **$P < 0.01$). (C) Endonuclease G (green color) translocation levels and nuclear areas (Hoechst 33258) were analyzed by 2.5D (pseudo-3D) in Zen 2009 software in cells treated with ETO and z-VAD.

Supplementary Movie S1. Control, DMSO-, doxorubicin (DOX)- or etoposide (ETO)-treated cells were observed by a real-time live-cell imaging system (related to Supplementary Figure S2).
